# Combinatorial investigation of spin-orbit materials using spin Peltier effect

**DOI:** 10.1038/s41598-018-34493-6

**Published:** 2018-10-30

**Authors:** Ken-ichi Uchida, Michiko Sasaki, Yuya Sakuraba, Ryo Iguchi, Shunsuke Daimon, Eiji Saitoh, Masahiro Goto

**Affiliations:** 1 Research Center for Magnetic and Spintronic Materials, National Institute for Materials Science, Tsukuba, 305-0047 Japan; 2 Research and Services Division of Materials Data and Integrated System, National Institute for Materials Science, Tsukuba, 305-0047 Japan; 3 Department of Mechanical Engineering, The University of Tokyo, Tokyo, 113-8656 Japan; 4 Center for Spintronics Research Network, Tohoku University, Sendai, 980-8577 Japan; 5 Center for Green Research on Energy and Environmental Materials, National Institute for Materials Science, Tsukuba, 305-0047 Japan; 6 PRESTO, Japan Science and Technology Agency, Saitama, 332-0012 Japan; 7 Institute for Materials Research, Tohoku University, Sendai, 980-8577 Japan; 8 Advanced Institute for Materials Research, Tohoku University, Sendai, 980-8577 Japan; 9 Department of Applied Physics, The University of Tokyo, Tokyo, 113-8656 Japan; 10 Advanced Science Research Center, Japan Atomic Energy Agency, Tokai, 319-1195 Japan

## Abstract

Conversion between spin and charge currents is essential in spintronics, since it enables spin-orbit-torque magnetization switching, spin-current-driven thermoelectric generation, and nano-scale thermal energy control. To realize efficient spin-charge conversion, a variety of mechanisms, including spin Hall effects, Rashba-Edelstein effects, and spin-momentum locking in topological insulators, have been investigated and more comprehensive material exploration is necessary. Here we demonstrate high-throughput screening of spin-charge conversion materials by means of the spin Peltier effect (SPE). This is enabled by combining recently-developed SPE-imaging techniques with combinatorial materials science; using a composition-spread alloy film formed on a magnetic insulator, we observe the SPE-induced temperature change due to the spin Hall effect and obtain a continuous mapping of its composition dependence from the single sample. The distribution of the SPE signals reflects local spin-charge conversion capability in the alloy owing to unique heat-generation nature of the SPE. This combinatorial approach will accelerate materials research towards high-performance spintronic devices.

## Introduction

The spin-charge conversion is typically realized by the direct and inverse spin Hall effects^1–4^ in a conductor with spin-orbit interaction. The direct spin Hall effect (DSHE) refers to the conversion of a charge current into a transverse spin current. An important application of the DSHE is the manipulation of magnetization dynamics; when a charge current is applied to a paramagnetic metal (PM)/ferromagnet or ferrimagnet bilayer systems with PM being a spin-orbit material, the DSHE in PM gives rise to spin accumulation near the interface and exerts torques on the magnetization of the ferromagnetic or ferrimagnetic layer, thus enabling the magnetization switching in spintronic devices, such as spin-transfer-torque magnetic random-access memories (STT-MRAMs)^5–9^. In contrast, the inverse spin Hall effect (ISHE) refers to the conversion of a spin current into a transverse charge current, enabling electric detection of spin currents. The DSHE (ISHE) is essential also in the field of spin caloritronics^10,11^, since it is used for exciting (detecting) thermo-spin effects; recent spin-caloritronics studies open up the potential of thermo-spin effects for versatile thermoelectric generators^12–15^ and nano-scale thermal energy controllers^16–23^. To develop the above applications, a great deal of experimental effort has been devoted to finding new materials with high spin-charge conversion efficiency^1–4^. However, detailed characterization of spin-charge conversion properties requires time-consuming experiments, which limits chance of discovering excellent materials. Therefore, preliminary screening methods for spin-orbit materials are highly desired in spintronics.

**Figure 1:**
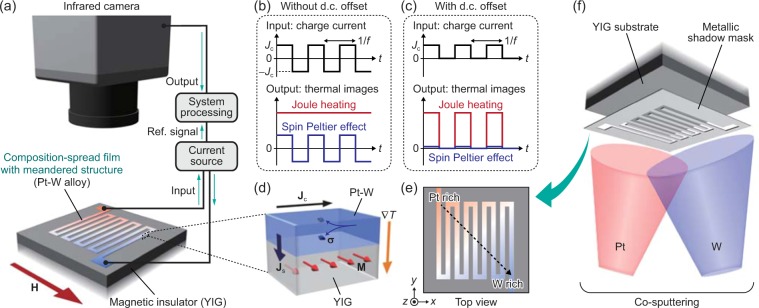
Combinatorial investigation of DSHE-driven SPE. (**a**) Schematic illustration of the LIT measurements and sample system used for measuring the SPE. The sample comprises a composition-spread Pt-W film with meandered structure formed on an YIG substrate. **H** denotes the magnetic field vector. (**b**,**c**) LIT conditions for the SPE (**b**) and Joule-heating (**c**) measurements. In the SPE (Joule-heating) measurements, a square-wave-modulated a.c. charge current with zero offset (finite d.c. offset *J*_c_/2), amplitude *J*_c_ (*J*_c_/2), and frequency *f* is applied to the Pt-W film. (**d**) The SPE induced by the DSHE near the Pt-W/YIG interface. **M**, **J**_c_, **J**_s_, and ∇*T* denote the magnetization vector of YIG, charge current applied to the Pt-W film, spatial direction of the spin current with the spin-polarization vector **σ** generated by the DSHE in Pt-W, and temperature gradient appearing as a result of the SPE-induced heat current, respectively. (**e**) Schematic illustration of the Pt-W/YIG sample from a top view. (**f**) Schematic procedure for fabricating the composition-spread Pt-W film on the YIG substrate.

As a probe of the spin-charge current conversion, we focus on the spin Peltier effect (SPE)^16–23^ driven by the DSHE in a junction comprising a PM film and a ferrimagnetic insulator (FI). Here, the SPE generates a heat current in linear response to spin-current injection. In the PM/FI junction, a charge current **J**_c_ applied to PM induces a transverse spin current via the DSHE, and this spin current then induces a heat current **J**_q_ across the PM/FI interface via the SPE, which satisfies the following symmetry:

1 $${{\bf{J}}}_{{\rm{q}}}\propto ({\boldsymbol{\sigma }}\cdot {\bf{M}}){{\bf{J}}}_{{\rm{s}}}\propto {\theta }_{{\rm{S}}{\rm{H}}}({{\bf{J}}}_{{\rm{c}}}\times {\bf{M}})$$

where **M**, **J**_s_, **σ**, and* θ*_SH_ denote the magnetization vector of FI, spatial direction of the spin current induced by the DSHE in the direction normal to the PM/FI interface, spin-polarization vector of the spin current, and spin Hall angle, respectively, and the DSHE follows **J**_s_ ∝ *θ*_SH_ (**σ** × **J**_c_) (Fig. 1(d))^17,19,22^. Therefore, by measuring the SPE-induced temperature change near the interface, one can obtain information on the magnitude and sign of the DSHE in PM. The DSHE-driven SPE have been observed by means of electrical measurements using thermocouple sensors^16,20^ and active infrared emission microscopy based on the lock-in thermography (LIT)^17–19,21–25^. Importantly, the LIT technique can visualize the spatial distribution of temperature change due to the DSHE-driven SPE with high temperature and spatial resolutions.

In this work, we have developed a technique enabling systematic and high-throughput screening of spin-orbit materials by means of the LIT-based SPE measurements. For high-throughput screening of physical properties, one often relies on combinatorial thin film deposition techniques^26–30^, which make it possible to fabricate a composition-spread film on a single substrate. By using the combinatorial material exploration together with the LIT technique, we have mapped the spatial distribution of the SPE signals in a composition-spread PM film formed on a single FI substrate. Since this method allows us to generate combinatorial libraries for the DSHE-driven SPE in a more efficient, reliable, and systematic way than conventional measurements, it will accelerate exploration of spin-orbit materials.

## Results

### Combinatorial investigation of SPE based on LIT

The combinatorial measurement procedures for the DSHE-driven SPE are schematically shown in Fig. 1. As a model system, we choose a hybrid structure consisting of a composition-spread paramagnetic Pt-W alloy film and a ferrimagnetic yttrium-iron-garnet (YIG) substrate. Although the Pt/YIG (W/YIG) junction is typically used for measuring the SPE^17,19,20,22^ because of the relatively-large positive (negative) spin Hall angle of Pt (W), the spin-charge conversion capability in Pt-W alloys has not been systematically investigated so far. The composition-spread Pt-W film was fabricated on an YIG substrate and patterned into meandered structure by co-sputtering Pt and W through a metallic shadow mask (see Fig. 1(f) and Methods for details), where the longitudinal direction of each wire of the meandered structure is along the *y*-direction. As depicted in Fig. 1(e), the top left (bottom right) corner of the composition-spread film is designed to be Pt-rich (W-rich). Owing to the meandered structure, the path of the charge current in the Pt-W film is well defined. To observe the SPE using the LIT technique, we measured the spatial distribution of infrared radiation thermally emitted from the sample surface while applying a square-wave-modulated a.c. charge current with zero offset, amplitude of *J*_c_ = 2.7 mA, and frequency of *f* = 25 Hz to the Pt-W film and extracted the first harmonic response of detected signals, where constant Joule-heating background (∝*J*_c_^2^) is excluded in the LIT thermal images (Fig. 1(b))^17–19^. Here, the detected infrared images are transformed into the lock-in amplitude and phase images by Fourier analysis and converted into temperature information through the calibration method used in our previous study^31^. When the magnetization of YIG is aligned along the *x*-direction and the charge current in the Pt-W film is along the *y*-direction, the SPE-induced heat current is generated along the *z*-direction across the Pt-W/YIG interface, resulting in the temperature change on the sample surface; this is the symmetry of the DSHE-driven SPE (Eq. (1)). Importantly, the SPE signals on the Pt-W/YIG interface reflect only the local temperature information without being affected by temperature broadening because of the presence of dipolar heat sources^17^. Therefore, the LIT measurements allow us to map the continuous composition dependence of the SPE signals reflecting the charge-to-spin current conversion properties of the Pt-W alloys in the single sample with high spatial resolution. Since all the data can be extracted from the same sample prepared under the same atmosphere, the composition dependence obtained by this method should be more reliable than that obtained from many separated samples.

### Characterization of composition-spread film

**Figure 2:**
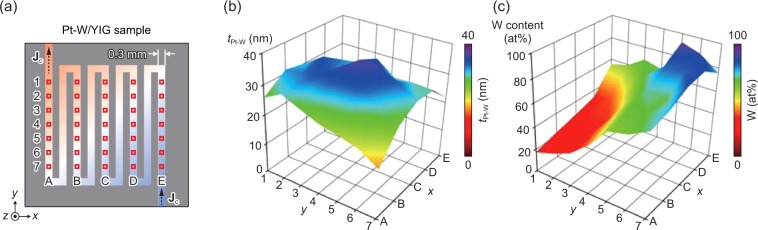
Thickness and composition distributions. (**a**) Schematic illustration of the Pt-W/YIG sample from a top view and definition of the areas A1-E7 on the meandered structure. (**b**,**c**) Distributions of the thickness *t*_Pt-W_ (**b**) and W content (**c**) of the composition-spread Pt-W film (see Methods for details).

In Fig. 2, we show the distribution of the thickness and composition of our combinatorial Pt-W film on YIG. Hereafter, the coordinates on the meandered structure in the *x*-direction (*y*-direction) are labeled with A, B, …, E (1, 2, …, 7) as shown in Fig. 2(a). For example, the top left (bottom right) area surrounded by a small square is referred as A1 (E7). The thickness and composition distributions, shown in this figure, and the temperature-modulation signals, shown later, are obtained from these areas. Figure 2(b) shows the thickness *t*_Pt-W_ distribution of the Pt-W film, measured with a stylus surface profilometer. The *t*_Pt-W_ value was observed to be varied from ~16 nm to ~37 nm, and its distribution is used to calibrate the charge-current density *j*_c_ in the film: *j*_c_ = *J*_c_/(*t*_Pt-W_ *w*_Pt-W_) with *w*_Pt-W_ (= 0.3 mm) being the width of the Pt-W wire. We also estimated the W-content distribution of the Pt-W film (see Methods for details). As shown in Fig. 2(c), the W content is varied almost as designed; the most Pt-rich (W-rich) area was observed to be A1 (E5) in which the composition is Pt_83_W_17_ (Pt_7_W_93_).

### Composition dependence of DSHE-driven SPE

**Figure 3:**
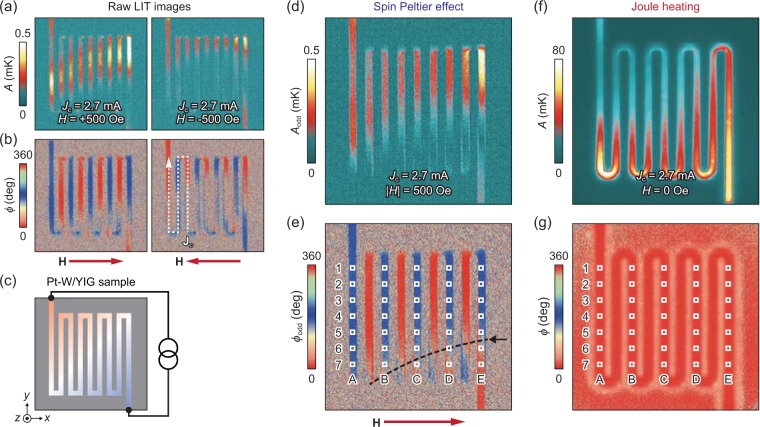
LIT images. (**a**,**b**) Raw amplitude *A* (**a**) and phase *ϕ* (**b**) images for the Pt-W/YIG sample at *J*_c_ = 2.7 mA and *f* = 25.0 Hz, measured in the SPE condition (see Fig. 1(b)). The left and right images were measured at the magnetic fields of *H* = +500 Oe and −500 Oe, respectively. (**c**) Schematic illustration of the Pt-W/YIG sample from a top view. (**d**,**e**) *A*_odd_ (**d**) and *ϕ*_odd_ (**e**) images for the Pt-W/YIG sample. The *A*_odd_ and *ϕ*_odd_ images are obtained by subtracting the raw LIT images at *H* = −500 Oe from those at *H* = +500 Oe and dividing the subtracted images by 2 (see the definition in the main text). (**f**,**g**) *A* (**f**) and *ϕ* (**g**) images for the Pt-W/YIG sample at *J*_c_ = 2.7 mA, *f* = 25.0 Hz, and *H* = 0 Oe, measured in the Joule-heating condition (see Fig. 1(c)).

Figure 3(a,b) shows the LIT amplitude *A* and phase *ϕ* images for the Pt-W/YIG sample, where the left (right) images were measured with applying the magnetic field of *H* = +500 Oe (−500 Oe) along the *x*-direction. We observed the clear temperature modulation depending on the charge-current direction on the Pt-W wires (compare the experimental results in Fig. 3(a,b) with the schematic illustration in Fig. 3(c)). Significantly, the distribution of the temperature modulation is changed by reversing the *H* sign, indicating the presence of the SPE contribution of which the sign is reversed in response to the magnetization reversal of YIG (see Eq. (1) and note that the raw images include *H*-independent background signals coming from the conventional Peltier effect because the Peltier coefficient of the composition-spread film varies from position to position). To extract the pure SPE contribution, we calculated the *A*_odd_ and *ϕ*_odd_ images showing the distribution of the current-induced temperature modulation with the *H*-odd dependence, where ${A}_{{\rm{odd}}}=|{A}_{+H}{e}^{-i{\varphi }_{+H}}-{A}_{-H}{e}^{-i{\varphi }_{-H}}|/2$ and ${\varphi }_{{\rm{odd}}}=-\,{\rm{\arg }}[({A}_{+H}{e}^{-i{\varphi }_{+H}}-{A}_{-H}{e}^{-i{\varphi }_{-H}})/2]$ with *A*_+*H*(-*H*)_ and *ϕ*_+*H*(-*H*)_ respectively being the *A* and *ϕ* values at *H* = +500 Oe (−500 Oe). As shown in Fig. 3(d,e), the *A*_odd_ and *ϕ*_odd_ distributions for the Pt-W/YIG sample are consistent with the feature of the DSHE-driven SPE; the temperature modulation appears only when the charge current is perpendicular to the magnetization of YIG. We found that the sign of the temperature modulation on the Pt-rich (W-rich) area is the same as that of the SPE signals for the Pt/YIG (W/YIG) system^17,19^ and observed clear sign change of the signals around the black dotted line in Fig. 3(e), where the *ϕ*_odd_ difference between E1-E5 and E6,E7 was observed to be ~180°.

**Figure 4:**
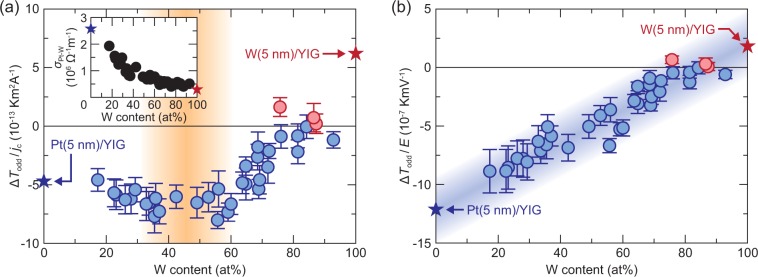
Composition dependence of DSHE-driven SPE in Pt-W/YIG. (**a**) W-content dependence of Δ*T*_odd_/*j*_c_ for the Pt-W/YIG sample. The data points are estimated from the *A*_odd_ and *ϕ*_odd_ values in Fig. 3(d,e) on the areas A1-E7, defined by the squares with the size of 10 × 10 pixels. The error bars represent the standard deviation of the data in the corresponding areas. The inset to (**a**) shows the W-content dependence of the electric conductivity *σ*_Pt-W_ of the Pt-W alloys. (**b**) W-content dependence of Δ*T*_odd_/*E* for the Pt-W/YIG sample, where *E* = *j*_c_/*σ*_Pt-W_ is the electric field in the Pt-W film. The blue (red) circle data points show the negative (positive) temperature-modulation signals for the Pt-W/YIG sample. The blue (red) star data points show the SPE signals for the Pt/YIG (W/YIG) system with the 5-nm-thick Pt (W) layer^19^ or electric conductivity values for the Pt (W) layer^23,44^.

Now, we are in a position to compare the distribution of the SPE signals (Fig. 3(d,e)) with that of the W-content distribution (Fig. 2(c)) for the Pt-W/YIG sample. In Fig. 4(a), we plot the W-content dependence of the SPE signal per unit charge-current density Δ*T*_odd_/*j*_c_ for the Pt-W/YIG sample on the areas A1-E7, where Δ*T*_odd_ ≡ *A*_odd_cos*ϕ*_odd_ shows the SPE-induced temperature modulation with sign information^21,22^ (note that the direction of the charge current is fixed on these areas and that the SPE signals observed in this condition do not contain the phase delay due to thermal diffusion^17,19,22^). The Δ*T*_odd_/*j*_c_ values systematically depend on the W content in the combinatorial Pt-W film; the magnitude of the SPE signals exhibits maximum at the W content ranging from 35 at% to 60 at% and its sign is reversed around 80 at% (Fig. 4(a)). The observed variation of the SPE signals is irrelevant to bulk properties of the YIG layer, because all the data are obtained from the single sample.

To further discuss the observed behavior of the DSHE-driven SPE, we measured the distribution of the electric conductivity *σ*_Pt-W_ of the Pt-W alloys, which is also realized by the LIT technique. In our sample, owing to the well-defined path for the charge current, the distribution of the temperature change due to Joule heating represents the resistance distribution of the Pt-W alloys. Although the Joule-heating signal is eliminated in the SPE measurements (Fig. 1(b)), it can be observed by applying finite d.c. offset to the square-wave a.c. current when the Joule-heating contribution is much greater than the SPE contribution (Fig. 1(c)). Figure 3(f,g) shows the LIT images due to the Joule-heating-induced temperature modulation for the Pt-W/YIG sample, where the lock-in frequency is fixed at the maximum value for our system, *f* = 25.0 Hz, to reduce the temperature broadening caused by thermal diffusion as much as possible^19,22^. By combining the Joule-heating distribution with the thickness and composition distributions, we obtained the relative variations of *σ*_Pt-W_ for the Pt-W alloys from the single sample. We also measured the local *σ*_Pt-W_ value only at the area A1 by the four-probe method as a reference to determine the absolute values of *σ*_Pt-W_. The inset to Fig. 4(a) shows the resultant W-content dependence of *σ*_Pt-W_ for the Pt-W alloys, indicating that *σ*_Pt-W_ monotonically decreases with increasing the W content. By using the *σ*_Pt-W_ values, we can obtain the electric field *E* (= *j*_c_/*σ*_Pt-W_) at each position of the sample.

In Fig. 4(b), we show the W-content dependence of the SPE signal per unit electric field Δ*T*_odd_/*E* for the Pt-W/YIG sample. Since Δ*T*_odd_ is proportional to the spin current generated by the DSHE, the Δ*T*_odd_/*E* and Δ*T*_odd_/*j*_c_ values are proportional to the spin Hall conductivity *σ*_SH_ (= *θ*_SH_*σ*_Pt-W_) and angle *θ*_SH_ in PM^1,2^, respectively (Eq. (1)); both the parameters are important to determine the spin-charge conversion performance. Surprisingly, the Δ*T*_odd_/*E* values in our Pt-W/YIG sample were found to vary almost linearly with the W content, where the correlation between the SPE signals and Pt-W composition also covers the pure Pt and W films on YIG. The systematic data observed here confirm the validity of the SPE measurements for composition-spread materials.

## Discussion

The combination of the LIT-based SPE measurements and combinatorial thin film deposition techniques is useful as a means for high-throughput screening of spin-orbit materials. The strong correlation in Fig. 4(b) indicates that the magnitude and sign of the DSHE-driven SPE in the Pt-W/YIG systems can be predicted only by the composition of the Pt-W alloys and, unfortunately, the binary alloys used in this study do not exhibit outstanding spin-charge conversion properties in comparison with pure Pt. However, if combinatorial films include compositions or crystal structures with huge *θ*_SH_ or *σ*_SH_ values, they should be identified as anomalies or peaks in the continuous mapping of SPE signals (note that the DSHE can be strongly dependent not only on material compositions but also on structural phases^6,7^). Once the anomalies or peaks are found, one can proceed to detailed experiments only for the specified materials to estimate the parameters related to the DSHE-driven SPE signals, such as the spin Hall angle and conductivity in PM, spin diffusion length in PM, spin mixing conductance^32–35^ at PM/FI interfaces, and interfacial thermal resistance^36–39^ (note that these parameters cannot be determined only by the SPE measurements). Therefore, by using the high-throughput screening method based on the LIT, one can investigate spin-charge conversion properties efficiently. Importantly, the same measurement procedures are applicable to multi-element compounds, although our demonstration was performed using simple binary alloys. This method will thus dramatically increase the chance of finding excellent spin-orbit materials, in comparison with conventional measurements, and contribute to the development of various spintronic applications, because the highly-efficient spin-charge conversion improves the performance of the magnetization switching in STT-MRAM devices^5–9^, thermoelectric generation utilizing the spin Seebeck effect^12–15^, and thermal management utilizing the SPE^16–23^. The performance of our method will be further improved in combination with other high-throughput material characterization techniques, such as thickness, composition, and crystal-structure mapping methods.

Another significance of the high-throughput screening method based on the LIT is its versatility. This method is applicable to investigating not only the DSHE but also different spin-charge conversion mechanisms, e.g., Rashba-Edelstein effects^40,41^ and spin-momentum locking in topological insulators^42^. Furthermore, in addition to the SPE, the same measurement procedures can be used directly for combinatorial investigations of various thermoelectric phenomena, e.g., the recently-observed anisotropic magneto-Peltier effect^31,43^ and anomalous Ettingshausen effect^23,44,45^ in magnetic materials. This combinatorial approach can also be extended to various measurements including deposition condition, interface quality, and thickness dependences, which may provide detailed information about conversion properties between spin, charge, and heat currents. As the LIT measurements enable rapid accumulation of vast amounts of data on thermo-spin and thermoelectric effects, its performance will be maximized in combination with materials informatics^15,46^, which is helpful for uncovering the correlation between the accumulated data and physical parameters and for designing new materials and optimum structures. Therefore, we anticipate that the high-throughput screening method developed in this work will be a driving force to accelerate basic and applied research in spintronics, spin caloritronics, and thermoelectrics.

## Methods

### Fabrication and characterization of composition-spread Pt-W film

The single-crystalline YIG (111) was grown on a single-crystalline Gd_3_Ga_5_O_12_ (111) substrate with a thickness of 0.48 mm by Shin-Etsu Chemical Co., Ltd. by means of a liquid phase epitaxy method. Here, to improve the lattice matching between YIG and Gd_3_Ga_5_O_12_, a tiny amount of Y in YIG is substituted by Bi. The YIG/Gd_3_Ga_5_O_12_ substrate was cut into the 10 × 10-mm^2^ square shape. The YIG surface was mechanically polished with alumina slurry and the thickness of the YIG layer after polishing was observed to be 35 μm. The composition-spread Pt-W film was then fabricated on the polished YIG surface and patterned into meandered structure by co-sputtering Pt and W through a metallic shadow mask, where the deposition of the Pt and W films were performed by means of an rf magnetron method using a combinatorial sputter coating system^29,30^. To obtain a steep composition gradient in the small substrate, the diameter of the Pt and W sputtering beams is reduced to 2 mm near the substrate and the center of the deposited area for Pt is deviated from that for W by ~5 mm on the substrate, where the deposited area for each material spreads over ~7 mm in the diameter. The width of the Pt-W wire is 0.3 mm and the distance between the centers of the adjacent wires is 0.6 mm. The thickness of the Pt-W film at the areas A1-E7, defined in Figs 2(a) or 3(e,g), was measured with a stylus surface profilometer (KLA-Tencor, Alpha Step D-120). The composition distribution of the Pt-W film was determined as follows. In addition to the Pt-W/YIG sample used for the LIT measurements, we fabricated a Pt film and a W film separately on two YIG substrates under the same condition as used for the preparation of the composition-spread Pt-W film. Then, the thickness distribution of the Pt and W films was measured independently by using the surface profilometer. By combining the obtained thickness distribution with volumetric atomic density of Pt and W, we estimated the composition distribution of the Pt-W film. The results in Fig. 2(b,c) were obtained by linear interpolation of the measured thickness and composition values for the areas A1-E7.

We also investigated the position dependence of the crystal structure of the Pt-W film using x-ray diffraction (XRD) with Cu-*K*α radiation source. The beam size of x-ray was reduced to 300-μm diameter by a collimator. The out-of-plane XRD patterns measured at the different positions on the Pt-W film always show only one strong peak at 2*θ* = 39–40°, which seems to originate from the (111) peak of fcc Pt or the (110) peak of bcc W. Since this result suggests strong textured growth of our Pt-W film, it is difficult to determine the position where bcc-to-fcc transition occurs. However, we are able to conclude from this XRD measurement that Pt and W atoms are simply mixed in our Pt-W film with keeping original fcc/bcc structure without forming secondary compounds.

### Lock-in thermography measurements

In the LIT measurements, one inputs a periodic external perturbation, that is, spin-current injection in our experiments, to a sample and extracts thermal images oscillating with the same frequency as the perturbation. The obtained thermal images are transformed into the lock-in amplitude and phase images by Fourier analysis. Here, the phase image gives information about the sign of the temperature modulation depending on the periodic perturbation as well as the time delay due to thermal diffusion. In general, the temperature broadening due to thermal diffusion is suppressed with increasing the frequency of the input perturbation^17,19,22^. By contrast, the temperature modulation induced by the SPE is known to reach a steady state within a very short time and has no lock-in frequency dependence in the frequency range used in this study^17,19,22^. During the LIT measurements, an in-plane magnetic field **H** with a magnitude of *H* = ±500 Oe was applied along the *x*-direction to align the magnetization of YIG along the **H** direction. To enhance infrared emissivity and to ensure uniform emission properties, the surface of the Pt-W/YIG sample was coated with insulating black ink, the emissivity of which is >0.95, commercially available from Japan Sensor Corporation. All the measurements were carried out at room temperature and atmospheric pressure.

## Data Availability

The data that support the findings of this study are available from the corresponding authors upon reasonable request.
